# Herpes Simplex Virus Infection in Neonates Born to Asymptomatic Mothers: A Case Series

**DOI:** 10.7759/cureus.32393

**Published:** 2022-12-11

**Authors:** María José Sánchez Pujol, Alexandre Docampo Simon, Lucía Sanguino, Mar Blanes, Isabel Betlloch

**Affiliations:** 1 Servicio de Dermatología. Alicante Institute for Health and Biomedical Research (ISABIAL), Hospital General Universitario de Alicante, Alicante, ESP; 2 Servicio de Pediatría. Alicante Institute for Health and Biomedical Research (ISABIAL), Hospital General Universitario de Alicante, Alicante, ESP; 3 Department of Clinical Medicine, Miguel Hernandez University of Elche, Alicante, ESP

**Keywords:** herpes simplex virus 1, herpes simplex virus 2, mother-to-child transmission, prevention, neonatal infection

## Abstract

Neonatal herpes is a rare condition and it is normally acquired through vertical transmission in the peripartum period. Delayed diagnosis and treatment of this condition are associated with high morbidity and mortality. We present five cases of neonatal herpes in infants born to asymptomatic mothers. Three of these infants were girls, three were born preterm, three were born after prolonged rupture of membranes, three had herpes simplex virus (HSV) type 2, and one had central nervous system (CNS) involvement. In all cases, the dermatologist played a key role in establishing an early diagnosis. Given the absence of a vaccine or a cost-effective method of screening for HSV infection in asymptomatic mothers, the current management strategies focus on the prevention of maternal infection and mother-to-child transmission, as well as early diagnosis and treatment of neonatal infection.

## Introduction

Neonatal herpes is a rare condition, and it is mostly acquired (85%) through vertical transmission in the peripartum period. The estimated annual incidence of neonatal herpes infection is between 1.6 and 8.4 per 100,000 live births [1]. The risk factors for vertical transmission include primary maternal infection, detection of herpes simplex virus (HSV) in the vaginal discharge, prolonged rupture of membranes, HSV-1 serotype, vaginal delivery (although elective cesarean delivery does not eliminate the risk of transmission since intrauterine infection is possible), and use of monitoring or instruments that can damage the skin barrier of the neonate [1,2,3].

HSV-1 infection may be asymptomatic in two-thirds of women, and 80% of neonates who become infected are born to mothers with no history of genital herpes [4]. Hence, early diagnosis of the condition requires a high index of suspicion [2]. Late diagnosis and treatment are associated with high morbidity and mortality [3,4]. In this report, we present five cases of neonatal herpes in infants born to asymptomatic mothers.

## Case presentation

Case 1

**Figure 1:**
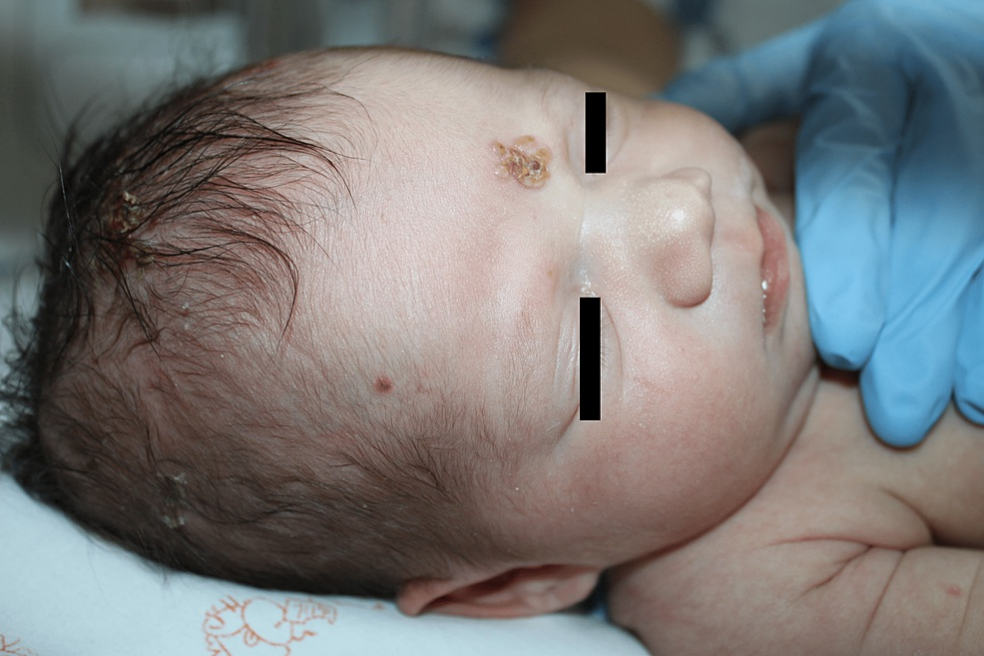
Case 1 Grouped vesicles on an erythematous base, bilaterally distributed on the forehead and scalp.

The first case was a girl born at term by vacuum-assisted vaginal delivery after prelabor rupture of membranes. At five days of life, she developed grouped vesicles on an erythematous base on her forehead and scalp (Figure 1). She was afebrile and in good general health. Physical, ophthalmologic, and neurological examinations revealed no further findings of interest. The polymerase chain reaction (PCR) test for HSV-1 was positive in the blood and the skin lesions and negative in the cerebrospinal fluid (CSF). Blood and CSF testing revealed normal results. A gynecological examination of the mother revealed no lesions; however, HSV-1 PCR of the vaginal discharge was positive. The newborn received intravenous acyclovir at 20 mg/kg every eight hours for two weeks, followed by oral acyclovir at 300 mg/m^2^ every eight hours for six months. She had no recurrence or sequelae.

Case 2

This infant was a girl born by unassisted vaginal delivery more than 24 hours after prelabor rupture of membranes, at 32+0 weeks of a dichorionic diamniotic pregnancy. At five days of life, she developed an erosive lesion on her right cheek, followed by vesicles and ulcers on her right cheek, philtrum, and upper lip. After collecting conjunctival, rectal, buccal, and cutaneous swabs, and CSF and blood samples, we started the infant on empiric intravenous acyclovir. HSV-2 PCR testing returned positive results in all the samples except the rectal swab and the infant was diagnosed with HSV-2 infection of the central nervous system (CNS). She later developed two corneal ulcers. The eye fundus examination, physical examination, blood test, imaging tests, and electroencephalogram showed no further abnormalities. The infant received intravenous and ophthalmic acyclovir for 21 days, after which we prescribed six months of oral treatment. Owing to social problems, a microbiological analysis of the mother was not performed.

Case 3

This infant was the twin sister of Case 2. She had no symptoms or skin lesions; however, given her epidemiological history, we requested microbiological tests. PCR for HSV-2 was positive in plasma and on the skin surface, but not in the CSF or the other peripheral samples. The physical examination, ophthalmologic examination, blood test, and imaging tests showed no abnormalities. The infant completed 14 days of intravenous treatment and started six months of oral acyclovir.

Case 4

**Figure 2:**
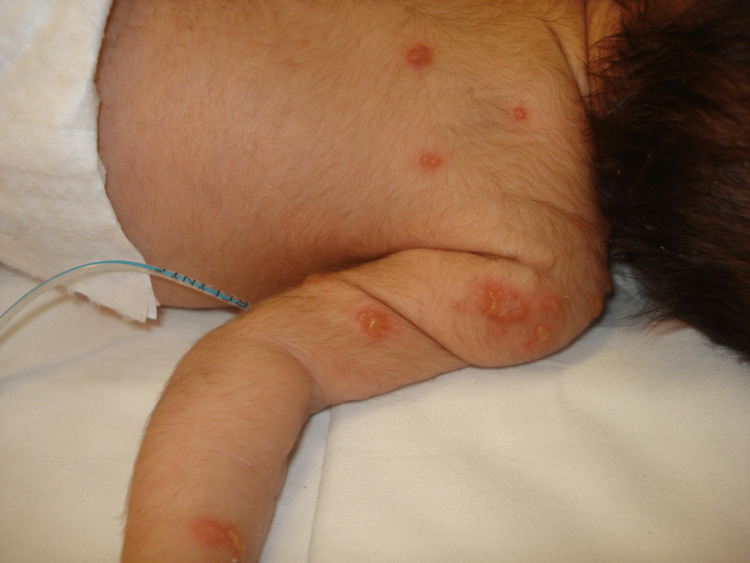
Case 4 Grouped vesicles on an erythematous base on the back and right upper limb

The fourth case was a boy born at 34+2 weeks by unassisted vaginal delivery, who was admitted to the neonatal ward with respiratory distress. His mother had a fever during labor. At 10 days of life, he developed grouped vesicles on an erythematous base on his upper back, right upper limb, and scalp (Figure 2). These lesions worsened and the infant became irritable, lethargic, and hypotonic. After requesting microbiological testing, we started him on intravenous acyclovir. A gynecological examination of the mother showed no lesions, but an immunoglobulin M (IgM) test for HSV-2 was positive. In the neonate, CSF HSV-2 PCR was negative, but serum HSV-2 IgM was positive. His general health improved and his skin lesions resolved, and the rest of the blood and imaging tests showed no abnormalities.

Case 5

**Figure 3:**
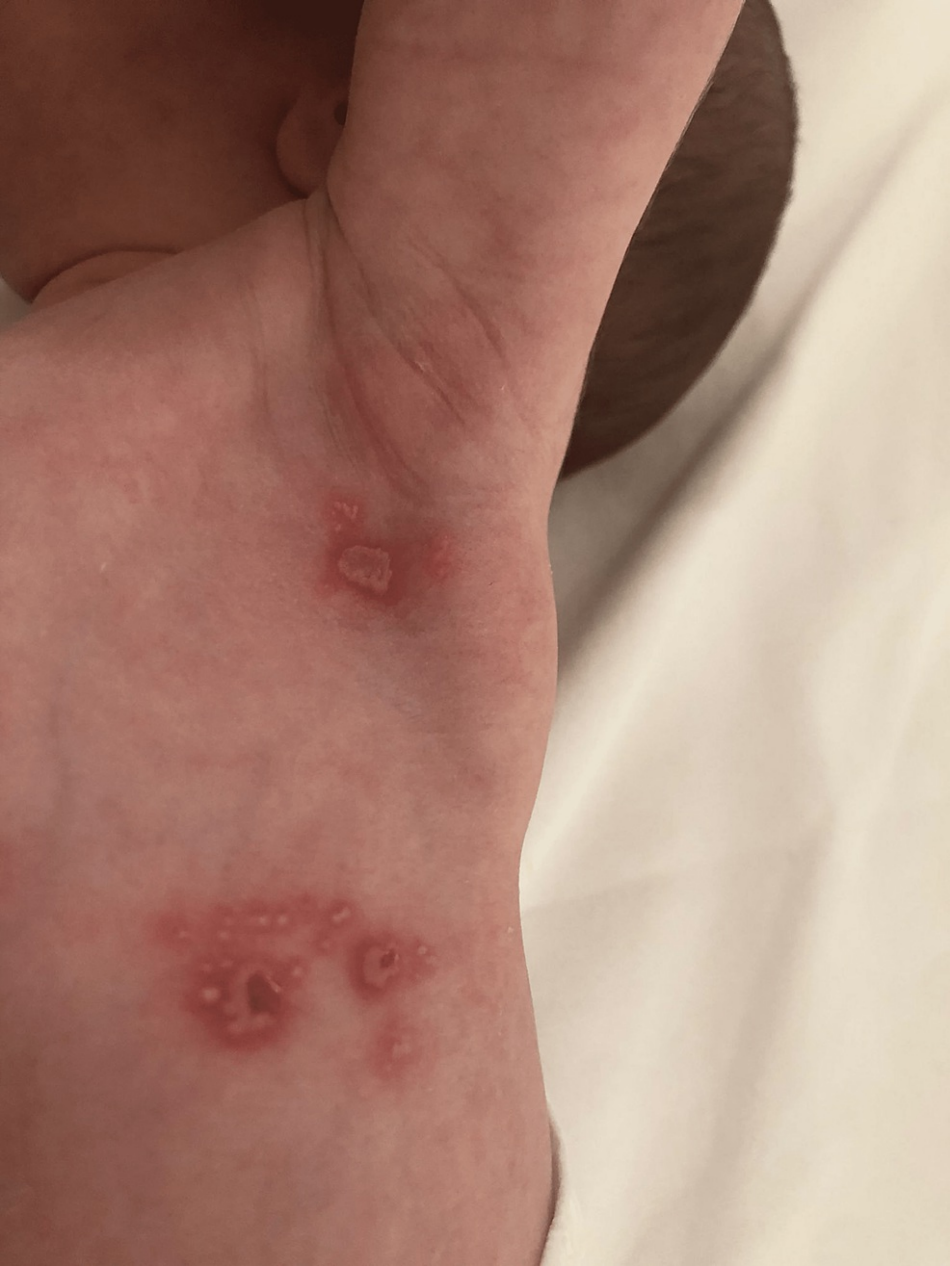
Case 5 Grouped vesicles on left armpit and abdomen

The final case was a boy born at term by vacuum-assisted vaginal delivery. At 10 days of life, he developed vesicles on an erythematous base on his abdomen, armpits (Figure 3), and scalp. He later developed vesicles on his right eyelid and buccal mucosa. He was afebrile and in good general health. Physical, ophthalmologic, and neurological examinations revealed no further findings of interest. PCR for HSV-1 was positive in the blood, conjunctiva, rectal mucosa, and skin lesions but negative in CSF. The infant completed 14 days of intravenous treatment and started six months of oral acyclovir.

Table 1 summarizes the clinical data of the five infants with neonatal herpes.

**Table 1:** Clinical and epidemiological characteristics of five cases of herpes simplex infection in neonates born to asymptomatic mothers with no history of genital herpes CSF: cerebrospinal fluid; CNS: central nervous system; F: female; h: hours; GAB: gestational age at birth; HSV: herpes simplex virus; IgM: immunoglobin M; M: male; microbiol. anal.: microbiological analysis; PCR: polymerase chain reaction; PROM: prolonged rupture of membranes; RUL: right upper limb; wk+d: weeks+days

Case	Sex	GAB (wk+d)	Delivery	PROM	Microbiol. anal. (mother)	Onset of lesions	Location of lesions	Involvement	Serotype	Microbiol. anal. (neonate)
1	F	39+4	Vaginal, assisted	>10 h	PCR +: vaginal discharge	Day 5	Forehead, scalp	Skin/mucosa	HSV-1	PCR +: blood, skin
2	M	32+0	Vaginal, unassisted (1st twin)	>24 h	Not performed	Day 5	Cheek, philtrum, upper lip	CNS	HSV-2	PCR +: blood, skin, conjunctiva, mouth, CSF
3	F	32+0	Vaginal, unassisted (2nd twin)	>24 h	Not performed	No	No	Skin/mucosa	HSV-2	PCR +: blood, skin
4	M	34+2	Vaginal, unassisted	No	IgM +	Day 10	Upper back, RUL, scalp	Skin/mucosa	HSV-2	IgM +
5	M	40+4	Vaginal, assisted	No	Not performed	Day 10	Scalp, armpits, abdomen	Skin/mucosa	HSV-1	PCR +: blood, skin, conjunctiva, rectal mucosa

## Discussion

Neonatal herpes transmission can occur in the uterus (5%), during the perinatal period (85%), or during the postnatal period (10%) [5,6]. The most common clinical form involves the skin, eyes, and mouth (45%) and manifests in the first 10-12 days of life as vesicles or ulcers on the skin, sometimes accompanied by buccal or ophthalmic lesions. HSV infection can also affect the CNS. This form can develop up to six weeks after birth, and the signs include irritability, lethargy, refusal to eat, hypothermia or fever, bulging fontanel, and focal or generalized convulsive seizure, sometimes accompanied by skin lesions (35%) [5,6]. The most severe form of neonatal herpes is disseminated infection, affecting various organs such as the skin, eyes, liver, lungs, nervous system, or adrenal glands, and manifesting as septic symptoms with multiorgan involvement.

In recent years, studies conducted in various countries have shown an increase in genital HSV-1 infection, associated with changes in sexual behaviors and viral pathogenicity [2,3]. In Spain, HSV-2 still predominates in genital herpes, though genital HSV-1 (which has a lower risk of recurrence) is becoming increasingly common in young women [2,4].

In our case series, 60% of the infants were girls, 60% were premature, and 60% were born after prolonged rupture of membranes. In all cases, delivery was vaginal and the mother had no lesions or history of genital herpes. The skin lesions observed in the neonates were grouped vesicles on an erythematous base, in different locations. HSV-2 infection predominated, and one infant had CNS herpes while the others had the skin/mucosal form (and also positive microbiological blood test results). As none of the mothers had a history of genital herpes, we initially considered other diagnoses (e.g., bullous impetigo). The dermatologist played a key role in the early diagnosis of HSV infection.

Most newborns exposed to HSV do not develop a disseminated infection, which calls into question the need for acyclovir treatment, given the association of this drug with neutropenia and nephrotoxicity in neonates [7]. However, the consequences of delaying treatment can be fatal in disseminated infections, and the risk-benefit ratio dictates that when there is a clinical suspicion of this form, we should start the infant on empirical treatment after taking several samples for microbiological analysis (including samples of the conjunctival, buccal, nasopharyngeal, and rectal surface; a swab or scrape from the cutaneous or mucosal lesions; and blood and CSF samples for HSV PCR). After confirming the infection, the tests we should request to determine which organs are affected include a blood test with complete blood count, coagulation, blood gases, and biochemistry (transaminases, total and direct bilirubin, electrolytes, urea, and creatinine); cytochemical analysis of CSF; an ophthalmological examination; and neuroimaging tests. Other imaging tests can be requested as per the infant’s clinical features. Acyclovir is administered intravenously at a dose of 20 mg/kg (or a renal function-adjusted dose) every eight hours for 14 days in the case of skin/mucosal herpes, or 21 days for disseminated or CNS infection, followed by oral acyclovir at 300 mg/m^2^ every eight hours for six months. The infant must be monitored for possible recurrence, sequelae, or drug-related adverse events [3,6,7].

To date, no cost-effective method of maternal HSV screening for reducing neonatal infection has been devised [3,4]. In addition, preventative antiviral therapy is not recommended [3,4], and a vaccine is still under development [8]. Therefore, the main management strategies for neonatal herpes focus on preventing maternal infection and mother-to-child transmission (actively looking for symptoms or lesions, performing cesarean delivery if there are active lesions at the time of birth, administering antiviral drugs for recurrences, etc.) and diagnosing and treating neonatal infection early.

## Conclusions

We presented five cases of neonatal herpes in infants born to asymptomatic mothers. Neonatal herpes is a rare condition, normally acquired through vertical transmission in the peripartum period. Most of the infected neonates are born to mothers with no history of genital herpes. Hence, early diagnosis requires a high level of suspicion, and the dermatologist can play a key role in identifying typical skin lesions. Late diagnosis and treatment of this condition are associated with high morbidity and mortality. Current management strategies focus on the prevention of maternal infection and mother-to-child transmission, as well as early diagnosis and treatment of neonatal infection.
